# How Might Indices of Happiness Inform Early Intervention Research and Decision Making?

**DOI:** 10.1007/s41252-022-00288-0

**Published:** 2022-10-03

**Authors:** Amarie Carnett, Leslie Neely, Meng-Ting Chen, Katherine Cantrell, Erin Santos, Shahla Ala’i-Rosales

**Affiliations:** 1 Faculty of Education, Educational Psychology, Victoria University of Wellington, Wellington, New Zealand; 2 Department of Educational Psychology, The University of Texas at San Antonio, San Antonio, TX USA; 3 Department of Educational Psychology and Counseling, National Taiwan Normal University, Taipei, Taiwan; 4 Department of Behavior Analysis, University of North Texas, Denton, TX USA

**Keywords:** Indices of happiness, Social validity, Early intervention, Caregiver-led intervention, Autism

## Abstract

**Abstract:**

**Objectives:**

The child-caregiver relationship is the foundation for which intervention occurs. Therefore, the acceptability of the intervention should be considered for both parties. Indices of happiness (IOH) have shown to be effective in assessing social validity and providing insight to improving interventions to promote better quality of life. However, to date, there is limited attention to the integration of IOH in very early caregiver-led intervention. The purpose of this study is to explore how researchers and clinicians might collect direct data on IOH to assess the acceptability of an intervention.

**Methods:**

Participants in this study included 4 children, ages 19–26 months old, identified as “at-risk” for autism, and their caregivers. Caregiver-led intervention focused on pairing, play, and following the child’s lead. IOH data was collected on both child and caregiver using 10 s partial-interval recording. Data analysis from the intervention is presented using three different approaches: pre/post-analysis on an individual level, pre/post-analysis on a dyad level, and during intervention as a primary dependent variable.

**Results:**

Variations were seen in levels of happiness, both on an individual level and dyad level. IOH for caregivers increased in relation as their fidelity increased but child IOH decreased as they acquired the targeted skill.

**Conclusions:**

Direct observation of happiness data is likely to provide valuable insight into participants perception of an intervention. And retrospective analysis may be a valuable tool for reflection and guidance and planning of future interventions.

**Supplementary Information:**

The online version contains supplementary material available at 10.1007/s41252-022-00288-0.

The notion of “social importance” is well documented in the origin of applied behavior analysis. In Skinner’s seminal text, Science and Human Behavior (1953), much attention was given to the idea that behavior analysis has the capacity to solve socially important problems. Wolf (1978) further elaborated on the importance of assessing social validity and the development of better measurement systems to help behavior analysts determine if their clients are happy during treatment. Since the call to evaluate social validity, behavior analysts have incorporated various measures to assess the social validity of interventions (Dillon & Carr, 2007; Ferguson et al., 2019) including indirect assessments (e.g., questionnaires; Ferguson et al., 2019; Reimers et al., 1992; Wacker et al., 1998), direct assessments, such as the effects of an intervention over time (e.g., Kennedy, 2002), and secondary behaviors (e.g., engagement, attention, and indices of happiness).

Behaviors, such as smiling, laughing, and approaching, may be a direct indication of enjoyment of an intervention (e.g., Dillon & Carr, 2007; Green and Reid, 1996; Green et al., 1997; Green and Reid, 1999a; Green and Reid, 1999b; Green et al., 2005; Lancioni et al., 2002; Lancioni et al., 2005; Vernon et al., 2012). These behaviors, termed indices of happiness (IOH), have emerged as a potentially informative means of evaluating the social validity of interventions. For example, Vernon et al. (2012) extended the analysis of the early work of Koegel et al. (1996) with directly observed, not rated, combined occurrence and duration measures of positive parent and child affect, engagement, and shared favorable affect. The results indicated that, in addition to skills acquisition increases, the use of socially embedded activities within the PRT procedure increased directly observed IOH measures of child, parent, and shared affect across all participants. Similarly, Arbogast and Fryling (2015) provided a brief assessment of IOH observed during a behavior analytic intervention for young children with autism. Two conditions were randomized to evaluate the effect on a child’s IOH. During the ABA condition, a therapist used common ABA-based instruction (e.g., differential reinforcement, prompting, redirection) and during the non-behavior analytic condition, a caregiver interacted with the child as they naturally would. For both children, IOH was higher in the ABA condition compared to the non-ABA condition. More recently, Thomas et al. (2021) evaluated happiness behavior during a functional analyses of problem behavior for children with autism. Finally, Ramey et al. (2022) replicated the procedures from Parsons et al. (2012) to identify and confirm individual mood indicators for children with autism. Findings of this study showed individualized IOH and unhappiness could be reliably defined and measured. Taken as a whole, these studies highlight the importance of using IOH measurement to help inform the social validity of behavioral interventions.

Although the findings of these studies highlight the importance of comparing happiness across conditions, they are few and limited in the progression of working toward a universal, meaningful approach to the measure of happiness. Specifically, happiness is subjective and likely shown differently across people and contexts (Dillon & Carr, 2007; Lancioni et al., 2005; Parsons et al., 2012; Ramey et al., 2022). And further, although progress has been made to improve upon the rigor of our measurement, there is still a need for addressing the current limitations of our assessment, such as the accuracy and validated methods of use (Park & Blair, 2019). Given the concerns regarding potential unhappiness and trauma in autism therapy, there is increased urgency and ethical obligation, both within and outside of the field of early intervention to assess affect during the intervention process (Autistic Self Advocacy Network, 2022). Thus, analysis is needed to understand how meaningful measurement may be developed, interpreted, evaluated, and utilized to improve the quality of life of the people we serve and to protect well-being and enhance short- and long-term happiness.

The aim of this study is to present a procedure for defining IOH within the context of a very early caregiver-led intervention. To our knowledge, other than Vernon, et al. (2012), no research has addressed IOH for children as young as infants and toddlers. Use of IOH data during natural change agent interventions is particularly significant, as the researcher and clinician must consider acceptability of the intervention on multiple levels (i.e., caregiver and child) and multiple dimensions (e.g., skills, affect). Using retrospective data collection and analysis, this study also aims to present options for IOH data analysis. Finally, the decision-making considerations as well as the strengths and limitations of IOH data collection will be discussed.

## Methods

### Participants

The participants in this study were a part of a larger study that focused on developing a very early intervention for infants and toddlers aged 6–36 months. The current study consisted of four children and their caregivers participated in this study. A child participant qualified for the study if they met the following criteria: (1) considered “at-risk” for autism (based on age, APSI and MCHAT-R scores and/or sibling status), (2) between 6 and 36 months of age at the start of the study, (3) English was the primary language spoken in the home, and (4) had caregiver consent to participate in the project. Additional criteria for caregivers included (1) access to a video conferencing device and (2) access to a stable internet connection. The definition for “at-risk” was determined based on age specific criteria. The participants each worked with their respective caregiver. The children’s and their parents’ demographic data are presented in Tables 1 and 2 respectively.

**Table 1:** Child demographic information

Participants	Age	Gender	Race	Criteria
Nate	1 yr:7 mos	M	Hispanic	At-risk for ASD due to sibling w/ ASD
Kyle	2 yr:2 mos	M	African American	At-risk for ASD due to MCHAT score of over 3
Matt	1 yr:3 mos	M	Hispanic	At-risk for ASD due to sibling w/ ASD
Kris	2 yr:2 mos	M	White	At-risk for ASD due to MCHAT score of over 3

**Table 2:** Caregiver demographic information

Child	Caregiver age	Caregiver gender	Caregiver race	Caregiver marital status	Caregiver level of education
Nate	31 yrs	F	Hispanic	Married	Bachelor’s degree
Kyle	24 yrs	M	African American	Single	Some college
Matt	36 yrs	F	Hispanic	Married	Associates degree
*Kris	27 yrs	F	White	Single	Some college

^*^Caregiver reported having an ASD diagnosis

### Procedures

#### Sessions

All sessions occurred during the COVID-19 pandemic and the researchers facilitated the sessions via telehealth. The caregiver and child participated from their homes, using the child’s toys, activities, and snacks. The coach conducted sessions from a university-based lab. Participants and their coach connected via Zoom® software. All sessions were video recorded using the record feature. Appointments were scheduled for 1 h, two times per week, for 15 weeks. Each appointment was structured into three components, a 5-min introduction with the coach and caregiver, a 5-min data probe, and caregiver coaching.

#### Interventionists

One trainer and a coach participated in this study. The trainer was a doctorate level Board Certified Behavior Analyst (BCBA-D) with 10 years of experience implementing parent training for children with ASD and 6 years of experience training others using telehealth technologies. The coach was a BCBA with a master’s degree and 4 years of experience implementing parent training for children with ASD.

#### Experimental Design

Researchers selected a non-concurrent multiple baselines across participants design. The lead researcher randomly assigned the caregivers-child dyads within the multiple-baseline design to either three, four, or five baseline data points.

#### Baseline

The coach provided caregivers with a copy of the caregiver fidelity rubric (see Appendix A) prior to the baseline phase. They then instructed the caregiver to “show us how you play with your child.” During the baseline, the coach observed but did not provide any instruction or feedback regarding the rubric or expected behaviors. Two to three baseline sessions were conducted per appointment with baseline lasting no less than two appointments.

#### Training

Following the completion of the baseline phase, the coach taught the caregivers how to implement each task listed on the caregiver fidelity rubric using teach-model-coach-review (e.g., Roberts et al., 2014). Each appointment started with a brief introduction during which the coach and caregiver discussed any issues relevant to the appointment. The coach then provided the caregiver with a copy of the caregiver fidelity rubric.

This introduction was followed by the 5-min data collection probe session. During the probe, the coach observed the caregiver engaging with their child without providing any feedback or direction and collected data on their fidelity of implementation using the caregiver fidelity rubric (Appendix A). After the probe session, the remainder of the session included teaching, modeling, and coaching the caregiver on how to implement the play sessions. The coach provided feedback based on the caregiver’s current level of responding.

#### Measures and Data Analysis

The primary data used to guide the evaluation of the intervention were the caregiver fidelity data and child social engagement. The IOH data were extracted for the purpose of the present analysis and, at the time, were not used as a primary indicator for intervention evaluation.

The researchers identified and operationalized IOH for both the caregiver and the child participant. The researchers identified six main IOH behaviors for the caregivers. The IOHs for caregivers included vocalized statements of praise, clapping, smiling, dancing, laughing/giggling, and elevated vocal pitch (see Table 3 for operational definitions).

**Table 3:** IOH operational definitions

IOH	Definition
Vocalized statements of praise	Any instance when participant verbalizes positive engagement. For example, saying “yay,” “you did it,” “good job,” “wow,” etc
Clapping	Any instance when both of client’s hands make contact, resulting in an audible noise that can be heard from at least 2 feet away. Excludes inappropriate behaviors such as hitting
Smiling	Any instance when the sides of the participant’s mouth curve upward; may or may not show teeth or open mouth
Dancing	Any instance when the participant makes short steps while swinging arms around, bounces up and down on the balls of the feet, or bends knees repeatedly in a rhythmic pattern
Laughing/giggling	Any instance when the participant’s mouth curves upward and shows teeth while in combination with short, repetitive vocalizations. May result in the participant’s shoulders moving up and down
Elevated vocal pitch	Any instance when a participant makes a high pitch audible noise from their mouth that can be heard from at least 2 feet away. Does not include elevated vocalizations that are accompanied by a distressed look or problem behavior such as throwing, avoiding the therapist or aggression

The child participants’ IOH were individually identified. During the intake process, researchers asked caregivers two questions: (1) what kinds of things does your child enjoy doing; (2) how do you know when your child is happy? The researchers also conducted an indirect reinforcer assessment interview (see Table 4 for the questions). Using the responses, researchers developed operational definitions of the child IOHs and asked the caregiver to confirm if the definitions were accurate. Researchers then validated the IOH through observation of the child with known preferred items or activities (Green & Reid, 1996). For Nate, happiness was defined as any instance of smiling or laughing (see Table 3 for operational definitions for IOH). For Kyle, happiness was defined as smiling, laughing, and dancing. For Matt, happiness was defined as smiling, laughing, and elevated vocal pitch. Finally, for Kris, happiness was defined as smiling, laughing, and elevated vocal pitch. Caregiver and child IOH data were measured using 10-s partial interval recording, within the 5-min sessions.

**Table 4:** Indirect reinforcer assessment interview

Questions
1. Some children really enjoy looking at things such as a mirror, bright lights, shiny objects, and TV. What are the things your child most likes to look at?
2. Some children really enjoy different sounds, such as listening to music, car sounds, whistles, beeps, sirens, clapping, and people singing. What are the things your child most likes to listen to?
3. Some children really enjoy different smells such as perfume, flowers, coffee, and pine trees. What are the thigs your child most likes to smell?
4. Some children really enjoy certain snack foods and beverages such as ice cream, pizza, juice, biscuits, and crackers. What are the things your child most likes to eat and drink? 5. Some children really enjoy physical play or movement such as being tickled, wrestling, running, dancing, swinging, and being pulled on a scooter. What activities does your child most enjoy? 6. Some children really enjoy touching things of different temperatures, cold things like snow or an icepack, or warm things like a hand warmer, or a cup containing hot tea or coffee. What activities like this does your child enjoy? 7. Some children really enjoy feeling different sensations such as splashing water in a sink, felling vibration against the skin, or the felling of air blowing on the face from a fan. What activities like this does your child enjoy? 8. Some children really enjoy it when others give them attention such as a big hug, a pat on the back, receiving applause, and being told they did a “good job.” What form of attention do you think your child most enjoys? 9. Some children really enjoy certain toys such as puzzles, toy cars, balloons, comic books, flashlights, and bubbles. What are some of your child’s favorite toys or objects? 10. What are other items or activities that your child really enjoys? 11. What are your child’s top six food/drink items? These items must be available during session 12. What are your child’s top six play items/sensory stimuli? These items must be available during session

In addition to indices of happiness, researchers collected data on caregiver fidelity of implementing the play sessions using the caregiver fidelity rubric (see Appendix A). Adapted from the Sunny Starts DANCE program (Ala'i-Rosales et al., 2013), the rubric lists three primary skill categories: pairing, play, and following the child’s lead. Researchers at REDACTED FOR REVIEW expanded the categories to include 11 specific tasks as part of the coaching program shown in Appendix A. Researchers collected data on the occurrence of opportunities (trials) for the caregiver to complete tasks during a 5-min session. Researchers scored trials as either completed, not completed, or not applicable. Mastery criteria was considered at 100% fidelity.

Researchers also collected data on child social engagement using partial interval recording. Researchers defined social engagement as the percentage of intervals in which the child engaged with the caregiver in play. Researchers defined *social engagement* as reaching to the caregiver, orienting body towards the caregiver, moving towards the caregiver, gazing towards the caregiver, and accepting stimuli from the caregiver. We excluded other forms of play, such as parallel play, when the child was engaged in play but not engaging with the caregiver.

#### Interobserver Agreement (IOA)

Raters collected IOA data for each dependent variable for a minimum of 33% of sessions within each phase for each participant (e.g., 33% of baseline, 33% of intervention sessions). Raters were trained by a lead researcher until they reached 100% reliability for at least one session. Interobserver agreement (IOA) was calculated for child occurrence or non-occurrence IOH using interval-by-interval agreement. The resulting IOA for child IOH averaged 99% (range, 97–100%) for Nate, 99.25% (range, 97–100%) for Kyle, 100% for Matt, and 100% for Kris. The resulting IOA for caregiver IOH averaged 91% (range, 83–100%) for Nate’s mother, 99.25% (range, 97–100%) for Kyle’s father, 99% (range, 97–100%) for Matt’s mother, and 99% (range, 97–100%) for Kris’ mother. The same method was used to calculate IOA for child social engagement. The resulting IOA for child IOH averaged 94% (range, 86–100%) for Nate, 97% (range, 93–100%) for Kyle, 93% (range, 86–100%) for Matt, and 97% (range, 93–100%) for Kris.

To calculate IOA for caregiver fidelity of the session, the lead author used exact agreement on the fidelity checklist. If both raters indicated that the caregiver completed or missed a certain step, the rater scored the step as an agreement. The author then divided the number of agreements by the total number of fidelity steps and multiplied by 100 to obtain a percentage. The resulting IOA for caregiver fidelity of implementation averaged 96% (range 92–100%) for Nate’s mother, 97.5% (range 92–100%) for Kyle’s father, 95% (range 88–100%) for Matt’s mother, and 98% (range 96–100%) for Kris’ mother.

#### Treatment Integrity

Researchers collected treatment integrity data for the coach’s adherence to the coaching fidelity rubric. The rubric shown in Appendix B consisted of 24 tasks subdivided into five categories: preparation, teaching, modeling, pre-session coaching, coaching during a session, and review. Treatment integrity data were collected for at least 30% of sessions. Treatment integrity was, on average, 97% (range, 90–100%) for the coach across sessions.

## Results

**Fig. 1:**
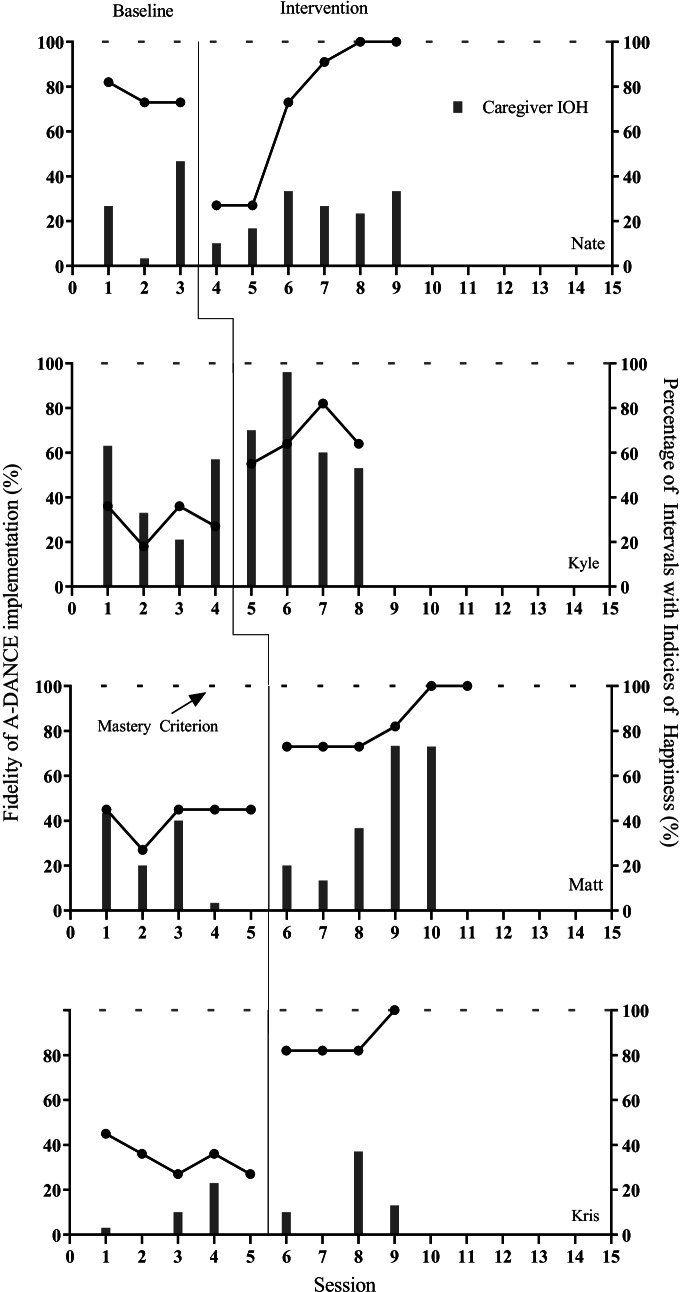
Caregiver fidelity of implementation and IOH

**Fig. 2:**
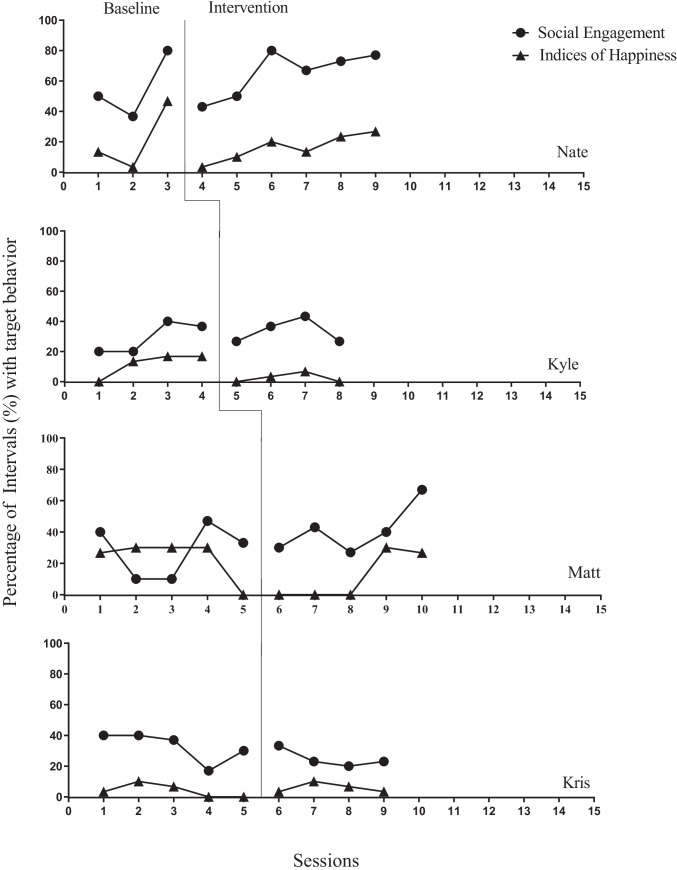
Percentage of intervals with child behavior for social engagement and IOH

To illustrate the different approaches to analyzing the IOH data, we will present three different analyses using the same data presented in Fig. 1 (caregiver dependent variables) and Fig. 2 (child dependent variables).

### Analysis 1: IOH as a Collateral Effect on an Individual Level

The first approach to analyzing IOH data is to consider IOH data as a collateral effect and analyze the data on an individual level. Starting with the caregiver outcomes, all of the caregivers improved their fidelity of implementation. Three of the caregivers reached mastery criterion in four or five sessions (Nate, Matt, and Kris). The fourth participant, Kyle, improved their fidelity of implementation from baseline average of 29% (range 18–36%) to 66% (range 55–82%) but did not reach 100% implementation fidelity (mastery criterion). When considering the caregiver IOH, three of the four participants (Kyle, Matt, and Kris) experienced increased IOH during intervention versus baseline levels. Kyle’s IOH increased from a baseline average of 44% (range 21–63%) to 70% (range 53–96%). Matt’s IOH increased from a baseline average of 21% (range 0–43%) to 43% (range 20–73%). Kris’s IOH increased from a baseline average of 7% (range 0–23%) to 15% (range 0–37%). The fourth participant, Nate, experienced a slight decreased in IOH from a baseline average of 26% (range 3–47%) to an average of 23% (range 10–33%), although Nate’s engagement in IOH did appear less variable in intervention versus baseline. Considering these results, one might conclude that the telehealth intervention was moderately effective in coaching caregivers to implement reciprocal play sessions and caregivers seemed to enjoy the telehealth sessions.

When looking on the child level, three of the four participants increased their social engagement above baseline levels. Nate increased social engagement from a baseline average of 56% (range, 37–80) to 65% (range, 43–80%). Kyle increased social engagement from a baseline average of 29% (range 20–40%) to 33% (range 27–43%). Matt increased social engagement from a baseline average of 28% (range 10–47%) to 41% (range 27–67%). The social engagement for the fourth participant, Kris, reduced from a baseline average of 33% (range 17–40%) to 25% (range 20–33%).

When considering the child IOH, all participants experienced decreased IOH during intervention versus baseline levels. Nate experienced a decrease in IOH from a baseline average of 21% (range 3–47%) to an average of 16% (range 10–37%). Kyle’s IOH decreased from a baseline average of 12% (range 0–17%) to 2.5% (range 0–7%). Matt’s IOH decreased from a baseline average of 23% (range 0–30%) to 11% (range 0–27%). Kris’s IOH decreased from a baseline average of 4% (range 0–7%) to 6% (range 3–10%). One might conclude that, although most of the participants improved their social engagement, none of the children appeared to enjoy the intervention based on their IOH.

### Analysis 2: IOH as a Collateral Effect on a Dyad Level

A second approach to analyzing IOH data is to consider IOH data as a collateral effect and analyze the data on a dyad level. Instead of analyzing it individually, the alignment of the IOH data between caregiver and child can be evaluated as a measure of *harmonious happiness* (Ala'i-Rosales et al., 2013)*.* The researchers calculated *harmonious happiness* by subtracting the caregiver IOH results from the child IOH results. The researchers then took the absolute value of the resultant. To analyze this data, we would want to see an absolute value of close to zero, indicating the child and caregiver were “happy” for approximately the same amount of time during the session. An increase in the absolute difference would indicate divergence in agreement and a decrease in absolute difference would indicate concurrence in agreement. For Nate, the average of the absolute value for IOH in baseline was 5 (range 0–13) and the average of the absolute value for IOH in intervention increased to 8 (range 0–13). For Kyle, the average of the absolute value for IOH in baseline was 31 (range 4–63) and the average of the absolute value for IOH in intervention increased to 67 (range 53–92). For Matt, the average of the absolute value for IOH in baseline was 12 (range 0–27) and the average of the absolute value for IOH in intervention increased to 31 (range 20–47). For Kris, the average of the absolute value for IOH in baseline was 7 (range 0–23) and the average of the absolute value for IOH in intervention increased to 14 (range 10–30). These results indicate that the IOH for the child and caregiver dyads diverged as the intervention progressed. If one of the goals of a play intervention is to increase child-caregiver relations, using this measurement of *harmonious happiness*, *that is*, *alignment of affect*, would suggest the telehealth coaching had a deleterious effect.

### Analysis 3: IOH as a Primary Outcome

The above analyses evaluated IOH as a collateral effect or a distal outcome. While this can help researchers to operationalize acceptability and validity of an intervention, researchers might consider using this IOH data as a primary outcome measure and base their data-based decision-making process on the results. For example, if we considered the caregiver IOH as the primary outcome variable, visual analysis (Kazdin, 2011) would suggest a negative effect for Nate, no effect for Kris, and small effects for Kyle and Matt. The single-case researcher would then have continued to collect data for all the participants until the IOH data improved and stabilized in the intervention phases. If reviewing the child data, the single-case researcher would have continued to collect intervention data or might have considered inserting a phase change and switching interventions to improve child IOH.

## Discussion

One of the more salient findings is that IOH for caregivers increased in relation as their fidelity increased but child IOH decreased as they acquired the targeted skill. There are several considerations for these conclusions. First, it is possible that IOH decreased during skill development, but, once they acquired the skills, IOH might have increased. Since the IOH did not improve for children, and they did not achieve steady responding in their targeted skill, the children were likely still in the acquisition phase. These findings support previous research identifying lower levels of IOH which are observed during work activities (Dillon and Carr, 2007; Yu et al., 2002). The one exception to this is Vernon et al. (2012). In this case, affect and synchronous engagement were identified as collateral effects of a play-based intervention specifically aimed at embedding social interactions during consequence delivery. In this case, favorable affect and synchronous engagement were viewed as a collateral effect of the training. As such, the current analysis and the Vernon et al. study have implications for how IOH data factor into interventions for very young children with autism. For example, if researchers and clinicians use IOH as a measure of treatment evaluation and decision making, it is possible that this will require longer intervention phases. Second, IOH data may be evaluated differently during skill acquisition, as some children may not enjoy a particular task that is foundational to learning. For example, Schatz et al. (2016) used video modeling to improve math instruction but not all children were reported to enjoy the task. While not all children enjoy learning math skills, it is an important skill for academic progression and future work can address methods to improve happiness. Relatedly, there may be methods to enhance the task time and increase social harmony between caregivers and children, such as embedding social interactions into consequence delivery (Vernon et al., 2012). Finally, it is also unclear if lower IOH rates are an indication that a task is unenjoyable or aversive. For example, expressing happiness for individuals with communication needs may be difficult to evaluate as the individuals cannot describe their private emotions (Charlop & Walsh, 1986; Parsons et al., 2012). In some cases, showing a lower level of IOH during an intervention may not be indicative of a child’s emotional state or that the intervention was aversive. Rather, perhaps both IOH and indices of sadness (IOS) might be an appropriate contrast to consider. More research is needed to develop procedures for individualizing measurement and for testing the reliability of behavioral indicators of happiness as well as unhappiness.

When considering the evaluation of happiness, this study did note improvements for three of the four caregivers. These results are intriguing as the results were immediate and the context of this study was during the early days of the COVID-19 pandemic (Her Majesty Queen, 2021). Research is beginning to document the extreme stressors that caregivers (Cluver et al., 2020), especially of children with disabilities (Chafouleas & Iovino, 2020), endured and continue to endure in response to the pandemic. As we extend our understanding of happiness as an outcome to be assessed, it may also be important to consider the dynamic interactions that occur between two individuals engaging during social interactions. That is, within a parent–child interaction, mutual enjoyment is a worthy goal in and one likely to lead to increased benefits. Evaluating IOH for participants *during* intervention may help researchers and services providers to better understand the social validity of an intervention and guide data-based decision making.

### Limitations and Future Research

These data are preliminary and collected via retrospective analysis (e.g., did not inform the intervention). However, retrospective analysis may be a valuable tool for reflection and guidance for future research. These data are also limited in that relatively few data points of IOH which were captured. Thus, it is not clear if a decreasing trend indicated dissatisfaction with the intervention or if the participants were experience a neutral state of IOH in the context of skill acquisition. Future research should include measurement of IOH and long-term analysis, especially when in the context of IOH is a secondary measurement.

Direct observation of happiness data is likely to provide valuable information regarding a participant’s perception of an intervention. This study contributes to that growing body of knowledge about the procedures and parameters of doing so. Clinicians and researchers might consider how to integrate this measure of social validity into early intervention for children with autism. This might be particularly important for caregiver-led interventions where the research and clinician must assess acceptability on both the caregiver and child level and when enhanced relationships are a goal in and of themselves.

## Supplementary Information

Below is the link to the electronic supplementary material.

Supplementary file1 (DOCX 17 KB)

## Declarations

All procedures performed in studies involving human participants were in accordance with the ethical standards of the institutional and/or national research committee and with the 1964 Helsinki Declaration and its later amendments or comparable ethical standards. Informed consent was obtained from all individual participants involved in the study. The researchers gained approval for this study from the University of Texas at San Antonio Institutional Review Board, #18–220.

## References

[CR1] Ala'i-Rosales S, Cermak S, Guðmundsdóttir K, Bondy A, Weiss MJ (2013). Sunny starts: DANCE instruction for parents and toddlers with ASD. Teaching social skills to people with autism: Best practices in individualizing interventions.

[CR2] Arbogast NR, Fryling MJ (2015). Brief assessment of indices of happiness during early and intensive applied behavior analysis. International Journal of Behavior Analysis and Autism Spectrum Disorders.

[CR3] Autistic Self Advocacy Network (2022).* Position statements: Autism research and therapies*. Retrieved from: https://autisticadvocacy.org/about-asan/position-statements/. Accessed 31 Jan 2022

[CR4] Chafouleas, S. M., & Iovino, E. A. (2020). *Initial impact of COVID-19 on the well-being of caregivers of children with and without disabilities*. Storrs, CT: UConn Collaboratory on School and Child Health.

[CR5] Charlop MH, Walsh ME (1986). Increasing autistic children’s spontaneous verbalizations of affection: An assessment of time delay and peer modeling procedures. Journal of Applied Behavior Analysis.

[CR6] Cluver L, Lachman J, Sherr L, Wessels I, Krug E, Rakotomalala S, Blight S, Hillis S, Bachman G, Green O, Butchart A, Tomilson M, Ward C, Doubt J, McDonald K (2020). Parenting in a time of COVID-19. The Lancet.

[CR7] Dillon CM, Carr JE (2007). Assessing indices of happiness and unhappiness in individuals with developmental disabilities: A review. Behavioral Interventions.

[CR8] Ferguson JL, Cihon JH, Leaf JB, Van Meter SM, McEachin J, Leaf R (2019). Assessment of social validity trends in the journal of applied behavior analysis. European Journal of Behavior Analysis.

[CR9] Green CW, Reid DH (1996). Defining, validating, and increasing indices of happiness among people with profound multiple disabilities. Journal of Applied Behavior Analysis.

[CR10] Green CW, Reid DH (1999). A behavioral approach to identifying sources of happiness and unhappiness among individuals with profound multiple disabilities. Behavior Modification.

[CR11] Green CW, Reid DH (1999). Reducing indices of unhappiness among individuals with profound multiple disabilities during therapeutic exercise routines. Journal of Applied Behavior Analysis.

[CR12] Green CW, Gardner SM, Reid DH (1997). Increasing indices of happiness among people with profound multiple disabilities: A program replication and component analysis. Journal of Applied Behavior Analysis.

[CR13] Green CW, Reid DH, Rollyson JH, Passante SC (2005). An enriched teaching program for reducing resistance and indices of unhappiness among individuals with profound multiple disabilities. Journal of Applied Behavior Analysis.

[CR14] Her Majesty Queen M (2021). Parents of children with disabilities and the COVID-19 pandemic. Developmental Medicine and Child Neurology.

[CR15] Kazdin, A. E. (2011). *Single-case research designs: Methods for clinical and applied settings* (2nd ed.). Oxford University Press.

[CR16] Kennedy CH (2002). The maintenance of behavior change as an indicator of social validity. Behavior Modification.

[CR17] Koegel RL, Bimbela A, Schreibman L (1996). Collateral effects of parent training on family interactions. Journal of Autism and Developmental Disorders.

[CR18] Lancioni GE, O'Reilly MF, Campodonico F, Mantini M (2002). Increasing indices of happiness and positive engagement in persons with profound multiple disabilities. Journal of Developmental and Physical Disabilities.

[CR19] Lancioni GE, Singh NN, O'Reilly MF, Oliva D, Basili G (2005). An overview of research on increasing indices of happiness of people with severe/profound intellectual and multiple disabilities. Disability and Rehabilitation.

[CR20] Park E-Y, Blair K-SC (2019). Social validity assessment in behavior interventions for young children: A systematic review. Topics in Early Childhood Special Education.

[CR21] Parsons MB, Reid DH, Bentley E, Inman A, Lattimore LP (2012). Identifying indices of happiness and unhappiness among adults with autism: Potential targets for behavioral assessment and intervention. Behavior Analysis in Practice.

[CR22] Ramey, D., Healy, O., & McEnaney, E. (2022). Defining and measuring indices of happiness and unhappiness in children diagnosed with autism spectrum disorder. *Behavior Analysis in Practice*, 1-16. 10.1007/s40617-022-00710-y 10.1007/s40617-022-00710-y PMC10050627 37006433

[CR23] Reimers TM, Wacker DP, Cooper LJ, De Raad AR (1992). Acceptability of behavioral treatments for children: Analog and naturalistic evaluations by parents. School Psychology Review.

[CR24] Roberts MY, Kaiser AP, Wolfe CE, Bryant JD, Spidalieri AM (2014). Effects of the teach-model-coach-review instructional approach on caregiver use of language support strategies and children’s expressive language skills. Journal of Speech, Language, and Hearing Research.

[CR25] Schatz RB, Peterson RK, Bellini S (2016). The use of video self-modeling to increase on-task behavior in children with high-functioning autism. Journal of Applied School Psychology.

[CR26] Skinner BF (1953). Science and human behavior.

[CR27] Thomas BR, Charlop MH, Lim N, Gumaer C (2021). Measuring happiness behavior in functional analyses of challenging behavior for children with autism spectrum disorder. Behavior Modification.

[CR28] Vernon TW, Koegel RL, Dauterman H, Stolen K (2012). An early social engagement intervention for young children with autism and their parents. Journal of Autism and Developmental Disorders.

[CR29] Wacker DP, Berg WK, Harding JW, Derby KM, Asmus JM, Healy A (1998). Evaluation and long-term treatment of aberrant behavior displayed by young children with disabilities. Journal of Developmental and Behavioral Pediatrics.

[CR30] Wolf MM (1978). Social validity: The case for subjective measurement or how applied behavior analysis is finding its heart. Journal of Applied Behavior Analysis.

[CR31] Yu DCT, Spevack S, Hiebert R, Martin TL, Goodman R, Martin TG, Harapiak S, Martin GL (2002). Happiness indices among persons with profound and severe disabilities during leisure and work activities: A comparison. Education and Training in Mental Retardation and Developmental Disabilities.

