# Supplementary material for: How Might Indices of Happiness Inform Early Intervention Research and Decision Making?
Source: Adv Neurodev Disord. 2022 Oct 3;6(4):567–76. doi: 10.1007/s41252-022-00288-0 (PMC9527140; doi:10.1007/s41252-022-00288-0)
Supplement: Supplementary file 1 — Supplementary file1 (DOCX 17 KB) [file 41252_2022_288_MOESM1_ESM.docx]

**Appendix A**

*Caregiver Fidelity Rubric*

| **Topic** | **Topic Skills** |
| --- | --- |
| **Pairing** | 1. No aversive stimuli is presented (e.g., demands, questions, work activities, etc.) |
|  | 2. Caregiver orients towards child at least 1 time every 30s |
|  | 3. Caregiver comments about play or materials the child is engaging with (at their  communication level) approximately every 30s |
|  | 4. Each child bid for engagement is matched by the caregiver within 3s |
|  | 5. Caregiver provides toys or activities to the child throughout the pairing session (e.g., give the  next train track, bounce the ball, hand over the food items) at a rate of at least 1 per min |
| **Play** | 6. Caregiver sits close enough to the child to engage in play |
|  | 7. Caregiver engages in parallel or cooperative play depending on child’s preference |
|  | 8. Caregiver models expansive play at least one time within the min (or 3 if entire session is  pairing) |
|  | 9. Caregiver models functional toy play at least one time during the min (or 2 if entire session is  pairing) |
| **Follow Child’s Lead** | 10. Caregiver matches child’s engagement in activity by engaging in activity or items based on  observed behaviors from child indicating continued engagement is preferred (e.g., indices of  happiness, handing items to caregiver, orientation towards caregiver, pointing, decreased  proximity to caregiver, etc.) within 10s |
|  | 11. Caregiver matches child’s disengagement in participation in activity by withdrawing or  changing activities contingent on behaviors indicating disengagement is preferred (e.g.  turning back, resisting sharing, verbally declaring “no”, increased proximity to caregiver etc.)  within 10s |

**Appendix B**

*Coaching Fidelity Rubric*

| **Topic** | **Topic Skills** |
| --- | --- |
| **Preparation** | 1. Coach ensures room is set up for the session (e.g., preferred items  environmentally arranged in boxes, up on shelves, etc.) |
|  | 2. Coach identifies target skill for the child |
|  | 3. Coach prints a copy of the rubric to provide the caregiver |
|  | 4. Coach starts the video recording, states the session number, date, participant  initials |
| **Teach** | 5. Coach provides session materials to caregiver (e.g., fidelity rubric) |
|  | 6. Coach vocally reviews session materials with caregiver |
|  | 7. Coach answers any questions caregiver has regarding the materials |
| **Model** | 8. Coach models target skill |
|  | 9. Coach uses caregiver-appropriate language that corresponds to the  handout to describe what they are modeling |
|  | 10. Coach models skills with 100% fidelity |
|  | 11. Explain the goal and its effects of the skill on the child |
|  | 12. If applicable, coach describe example and non-examples of the skill and  appropriate/non-appropriate times to target the skill (e.g. settings to  implement the skill, situations where not to implement) |
|  | 13. Coach answers any questions caregiver has regarding the materials |
| **Pre-Session Coaching** | 14. Coach provides feedback during role-play |
|  | 15. Questions by the caregiver for clarification are answered by Coach |
|  | 16. Coach asks caregiver if they are ready to implement |
|  | 17. Coach prompts caregiver at their current level of prompting as identified  by previous session data |
|  | 18. Descriptive feedback is given to caregiver for modifications to  implementation procedures when procedure is not implemented  with fidelity |
|  | 19. Behavior specific praise is used to encourage appropriate delivery of  instructional methods |
| **During Session** | 20. Coach starts the video recording, states the session number, date,  participant initials, and provides the caregiver with the SD “show us  how you play with your child” or something similar |
|  | 21. Coach allows the caregiver to play and teach their child for a 5 minute  session |
| **Review** | 22. Coach gives a verbal overview of each step of the implementation  procedure |
|  | 23. Coach asks if the caregiver has any questions |
|  | 24. Questions by the caregiver for clarification are answered by coach |
